# Sustainable Valorisation of End-of-Life Tyres Through Pyrolysis-Derived Recovered Carbon Black in Polymer Composites

**DOI:** 10.3390/polym17202771

**Published:** 2025-10-16

**Authors:** Dharanija Banala, Ylias Sabri, Namita Roy Choudhury, Rajarathinam Parthasarathy

**Affiliations:** Department of Chemical and Environmental Engineering, School of Engineering, RMIT University, Melbourne, VIC 3001, Australia; s4044729@student.rmit.edu.au (D.B.); ylias.sabri@rmit.edu.au (Y.S.)

**Keywords:** end-of-life tyres, pyrolysis, recovered carbon black, tyre char, polymer composites, sustainability

## Abstract

More than one billion end-of-life tyres (EOLTs) are produced worldwide every year, and this is continuously increasing and has become an issue in sustainable development. This review discusses recent developments in the management of EOLTs and focuses on pyrolysis, which produces valuable tyre-derived products (TDPs) like steel, gas, oil, and char. This review focuses on recovered carbon black (rCB), a refined char with great potential as a sustainable alternative to commercial carbon black (CB). The review introduces a novel classification system for CB, virgin carbon black (vCB), recovered carbon black (rCB), and sustainable carbon black (sCB) to guide the transition toward environmentally friendly materials. It also examines how rCB enhances polymer properties for addressing price volatility and reducing carbon footprint. Additionally, a SWOT analysis evaluates the strengths (cost-effectiveness, reduced environmental impact), weaknesses (quality consistency), opportunities (emerging markets, circular economy integration), and threats (competition from virgin materials) of using rCB as a polymer reinforcement. By positioning rCB as a key material, this review outlines pathways for addressing the EOLT crisis and advancing a circular economy.

## 1. Introduction

### 1.1. Global Tyre Market Analysis: From Market Growth to EOLTs Creation

**Figure 1:**
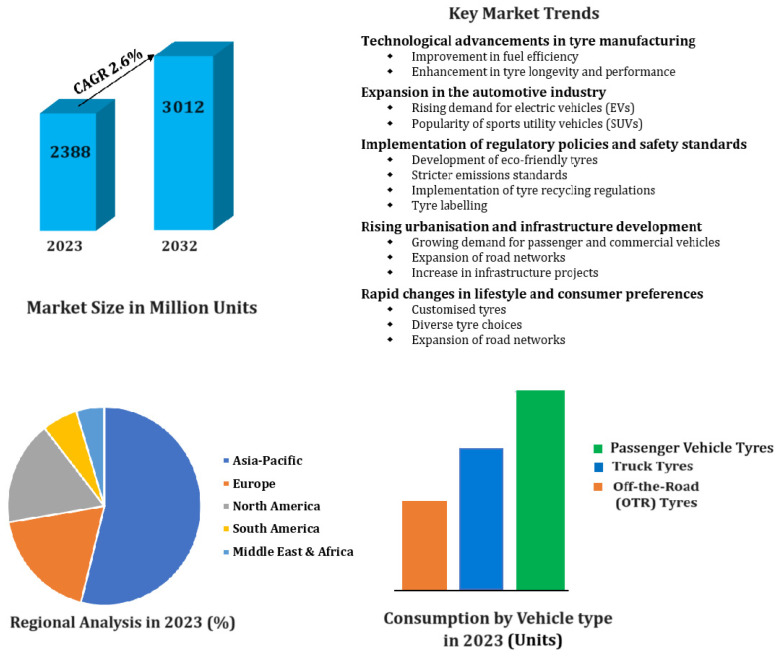
Outlook of the global tyre market, 2024–2032.

Vast vehicle usage contributes to the massive accumulation of waste rubber tyres worldwide each year [1]. As of 2023, the world market for tyres was 2388 million units, projected to reach 3012 million units in 2032, with a compound annual growth rate (CAGR) of 2.6% [2]. For example, in the fiscal year 2022–2023, Australia consumed more than 80 million new tyres [3]. The key drivers for the global tyre market include rapidly changing technology in manufacturing, strong automotive industry growth, strict regulatory policies, and growing consumer preference for specialty and green tyres. Figure 1 provides details and infographic data on the global tyre market over the forecast period to the year 2032 [2].

Tyres are hybrid products made from natural and synthetic rubbers, steel, carbon black, and fibres—including nylon, rayon, polyester, and adhesion promoters. However, the composition may vary depending on the manufacturer, type, and desired application. Tyres typically get into the environment by wearing out in various passenger vehicles, commercial trucks, and off-the-road (OTR) vehicles used in mining, agriculture, and other heavy-duty applications. A typical passenger vehicle tyre is expected to last 3.5 years, 1.5 years in a truck, and just about one year in OTR vehicles, depending on the mileage. When they wear out, they are classified as end-of-life tyres (EOLTs) or used tyres [4]. The 2024 tyre waste statistics show that more than one billion EOLTs are produced yearly [5]. Australia contributes over 56 million EOLTs each year, which is presumed to escalate over the following decade. Recent national studies have indicated that about 60–65% of all waste tyres end up in landfills or face other fates, such as dumping or illegal stockpiling, where little or no resource is recovered [4,6].

### 1.2. Navigating the Risks of EOLTs

The inappropriate handling of EOLTs can result in serious environmental and public health issues, of which the most significant are

1. Prolonged exposure of unused tyres to the environment could make them a breeding ground for insects, thus increasing vector-borne disease threats to human beings.
2. The open accumulation of waste tyres represents a potential fire risk in the event of ignition. In the case of fires, they release toxic gases, heavy metals, and hydrocarbons, leading to major pollution of soil, air, and groundwater [7].
3. The bulkiness and non-biodegradability of the waste tyres are additional burdens to landfill management because of the limitation on landfill space [8].

Therefore, EOLTs must be treated cautiously to protect the ecological system and human health. Although many strategies, such as reusing and recycling, along with traditional methods like landfilling and incineration, have addressed this challenge over the years, a persistent and effective solution is still being sought. The global commitment to reducing greenhouse gas emissions that cause global warming and climate change further complicates the issue. Additionally, the trend of increased volume of tyres in the global tyre market has made the accumulation of EOLTs inevitable, making the challenge even more significant.

### 1.3. Review Focus and Framework

This review explores different management pathways for EOLTs, with more attention to pyrolysis, an emerging approach. Furthermore, it presents an overview of pyrolysis or tyre-derived products (TDPs), such as scrap steel, syngas, oil, and char. Recovered carbon black (rCB) refers to refined char, free of wire and fabric and known for its semi-reinforcing properties [9]. This paper focuses on the reinforcing capacity of rCB to demonstrate its potential as a filler in polymer composites, identifying it as a valuable alternative to virgin carbon black (vCB).

To support this goal, the paper introduces a new classification of carbon black comprising virgin (vCB), recovered (rCB), and sustainable (sCB) carbon black.

1. Virgin carbon black (vCB): produced through the incomplete combustion of fossil fuel-derived petroleum feedstocks.
2. Recovered carbon black (rCB): generated from the pyrolysis of EOLTs, significantly reducing CO_2_ emissions by about 80% compared to vCB.
3. Sustainable carbon black (sCB): a forward-looking carbon black production produced from oil derived via advanced EOLT pyrolysis in a furnace reactor.

**Figure 2:**
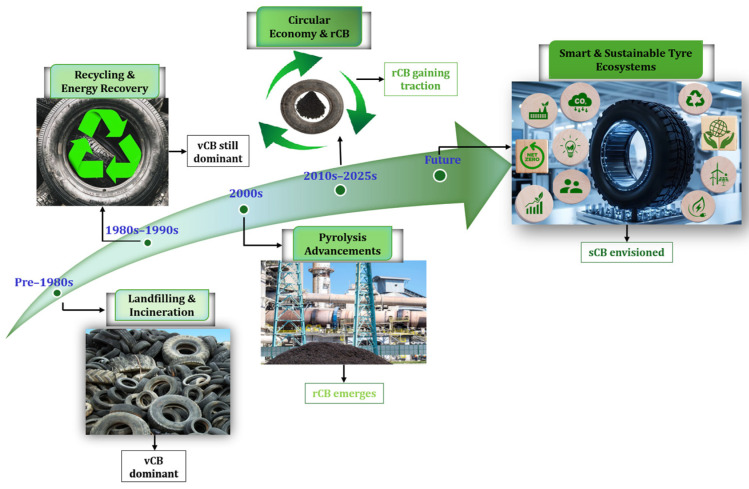
Progression of end-of-life tyres (EOLTs) strategies and carbon black evolution: from pre-1980s landfilling to cutting-edge recycling and sustainable carbon black.

The classification illustrates the evolution of EOLT management and suggests the future of tyre sustainability. Figure 2 highlights the history of EOLT management strategies, which have evolved alongside the use of carbon black. This transition has moved from outdated disposal methods to current sustainable practices. This evolution progresses from virgin carbon black (vCB) to recovered carbon black (rCB) and then to sustainable carbon black (sCB), paralleling the industry’s shift from extraction to recovery and finally to regeneration. The timeline is divided into five key phases:

- **Pre 1980s:** Landfilling and incineration dominated as disposal methods of EOLTs, with virgin carbon black (vCB) as the standard filler in tyre production.
- **From 1980s to 1990s:** Recycling and energy recovery practices emerged, but fossil-based vCB remained the primary material.
- **The 2000s:** Pyrolysis technology began to mature, producing rCB as a viable alternative and enabling the conversion of tyre waste into fuels and materials.
- **From 2010s to the present:** rCB gained traction globally, driven by circular economy policies, industrial partnerships, and urgent environmental goals. In Europe, the 2006 Extended Producer Responsibility (EPR) scheme significantly increased EOLT collection rates (up to 95% by 2019), creating reliable feedstock for pyrolysis. rCB production now plays a critical role in decarbonising the tyre value chain—reducing emissions from 10 kg CO_2_ per kg of vCB to only 2 kg CO_2_ for rCB. The EU’s July 2024 ban on carbon black imports from Russia further emphasised the strategic importance of local, sustainable alternatives like rCB.
- **Future:** Sustainable carbon black (sCB) is envisioned as the outcome of next-generation innovations in tyre recycling. These are superior pyrolysis units for emission-free material recycling, rubber devulcanization for full recyclability, and bio-based materials for replacing synthetics. Plus, recycling rubber is increasingly employed in high-performance uses, while AI-tracking and smart solutions facilitate closed-loop recycling and supply chain traceability.

The vCB–rCB–sCB classification encapsulates not only a material evolution but a broader shift toward environmentally responsible and technologically integrated tyre lifecycle management [10,11,12,13,14,15,16,17,18,19,20,21,22,23,24].

The review paper is structured in the following way: Section 2 discusses the various pathways towards addressing EOLTs, which include reuse, recycling, energy recovery, pyrolysis, and disposal. Section 3 discusses the pyrolysis process, influential parameters, scale-up issues, and technological advancement. Section 4 discusses the valorisation of pyrolysis products such as steel, syngas, oil, and char. Section 5 introduces the new carbon black classification, describing the differences between virgin (vCB), recovered (rCB), and sustainable (sCB) carbon black and their material properties. Section 6 examines the application of recovered carbon black (rCB) in polymer composites and its role as carbon-based reinforcement. Section 7 has a SWOT analysis to evaluate rCB as a sustainable filler in polymers by discussing its usage’s strengths, weaknesses, opportunities, and threats. SWOT analysis is a strategic planning tool to assess an organisation’s internal strengths and weaknesses and external opportunities and threats. It is pivotal in guiding decision-makers to make well-informed and effective decisions [25,26]. In this scientific context, SWOT is an established methodology for the technical, environmental, and application-based recovered carbon black (rCB) analysis. It identifies key research shortcomings such as standardisation issues, material performance challenges, and processing limitations. Additionally, it provides strategic guidance by promoting innovative areas in pyrolysis, composite design, and regulatory integration. This holistic approach enables a comprehensive evaluation of rCB across performance, sustainability, and scalability dimensions.

Finally, Section 8 concludes the review recommendations for future research directions focused on material standardisation, technological improvements, and commercial viability of rCB in support of a circular and sustainable tyre economy.

## 2. EOLT Management Pathways

**Figure 3:**
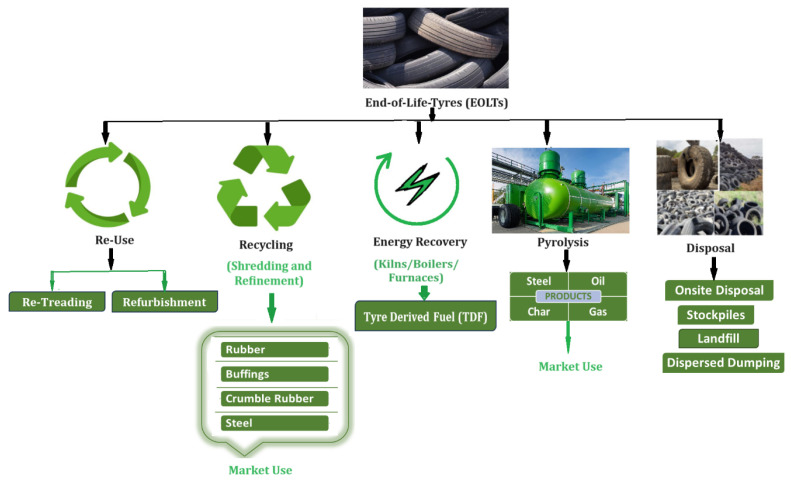
Management cycle of end-of-life tyres (EOLTs).

Many organisations, both public and private, are striving to improve the efficiency of EOLT management. Figure 3 shows an overview of common practices for managing EOLTs.

### 2.1. Re-Use

One common method for reusing scrapped tyres is retreading and refurbishing, which extends their operating life.

#### 2.1.1. Retreading

Retreading is a process that involves replacing the tread and sidewall materials of an old tyre with new ones, allowing the tyre to be returned to the market. This method offers significant benefits, including a 70% reduction in material usage, a 24% reduction in CO2 emissions, a 19% reduction in water use, and a 21% decrease in particulate air pollution compared to non-retreadable tyres. Truck tyres are usually designed for up to three retreads. Although retreading is widely used for truck tyres, it is not commonly used for passenger or OTR vehicles. However, retreading is not a foolproof solution for managing EOLTs because of the limitations on their lifespan and potential safety issues at high speeds [27,28].

#### 2.1.2. Refurbishment

This approach, which has been extensively used in the aviation industry, involves renewing tyres before they reach the end of service life. Refurbishment offers worn-out tyres a new life instead of discarding them. It renews the functionality, allowing them to be used repeatedly after being restored to a serviceable condition. The aviation industry is the best example of this practice. For instance, after a series of take-offs and landings, the tyres of large aircraft undergo refurbishment. Generally, these tyres can be refurbished up to seven times before they reach the end of their usable life due to their robust casing design, strict inspection protocols, and the controlled nature of their operating cycles [29].

### 2.2. Recycling

Recycling is an eco-friendly process of converting scrap tyres into useful materials for various applications. This typically involves traditional procedures like shredding, size reduction, and refining. These processes produce granulated rubber, tyre buffing, rubber crumb (or powder), and reclaimed steel [29]. In civil engineering applications, whole tyres are often utilised. The shredded tyres with sizes ranging from 25 to 300 mm are known as Tyre-Derived Aggregate (TDA). TDA has various uses, including fill-in material for road and rail foundations, drainage material, landfill construction, structure backfill, and insulation. Rubber granules and powder derived from tyres are used as the base materials in playground flooring, athletic tracks, and rubber-modified asphalt in road construction [28].

### 2.3. Energy Recovery

Energy recovery, also known as tyre-derived fuel (TDF) technology, is an effective method for managing EOLTs. High in calorific value, the EOLTs are an energy source for industrial kilns, boilers, and furnaces. This approach not only provides a solution for tyre disposal but also reduces the consumption of traditional fuels and minimises the environmental pollution associated with tyre dumping. However, the need to control emissions of harmful substances during this process is important. TDF is significantly more cost-effective than natural gas, coal, and petroleum coke, as well as in terms of the costs associated with the exploration, development, and transportation of these raw materials [13].

### 2.4. Pyrolysis

The pyrolysis process involves heating EOLTs in an inert atmosphere at a temperature typically ranging from 400 to 600 °C. It has emerged as an attractive technology for the management of EOLTs, with the potential for an environmentally friendly impact and the production of valuable products. However, the scale-up of pyrolysis plants faces limitations due to safety concerns, which will be discussed in the following section [30].

### 2.5. Disposal

#### 2.5.1. Stockpiles

Stockpiles are significant volumes or uncontrolled accumulations of EOLTs. When these stockpiles are large, they are typically considered illegal activities, especially compared to the small-scale dumping of just a few tyres. The disposal of scrap tyres often involves bundling them up and stacking them.

Management of legacy stockpiles of EOLTs presents several challenges; therefore, the approach to handling them should differ. Firstly, many owners consider these stockpiles as future assets rather than liabilities. Such perspectives overlook the inherent risks associated with stockpiles, such as fire risks and insect infestations. Consequently, the owners often leave the stockpiles in place until there is government action in the form of an abatement programme or regulatory enforcement. Secondly, ‘stockpile mapping’, which refers to assessing the actual quantity of EOLTs in stockpiles, is challenging to evaluate accurately.

#### 2.5.2. Landfill

Landfilling refers to the disposal of used tyres in a legally sanctioned landfill site approved by state environmental regulators. This method is sometimes preferred because collection fees charged by recycling companies can be higher than the municipal landfill costs. However, landfilling is not a sustainable strategy for handling EOLTs because leaching from tyres poses a significant environmental problem. The eco-toxic substances released through leaching have the potential to contaminate both water and soil, particularly threatening aquatic organisms. Therefore, landfilling is restricted in many developed nations, as it is considered the least favourable method for dealing with EOLTs. Several factors influence the pollution level from landfilling, including tyre size, soil permeability, and water contact time. Additionally, uncontrolled fires at landfills pose a significant risk to both the environment and human health [13,31].

#### 2.5.3. Onsite Disposal

Onsite disposal refers to the practice of discarding used tyres at the location of use. This method is commonly seen in the mining industry, OTR operations, and agriculture, often due to the higher costs associated with transporting the used tyre to a different location for disposal or recycling.

#### 2.5.4. Dispersed Dumping

Dispersed dumping refers to small, random waste disposal sites, often comprising multiple tyres. For example, waste may be abandoned along roadsides or in ravines in public and remote landscapes.

## 3. Pyrolysis of End-of-Life Tyres

Numerous methods have been employed to manage EOLTs, including retreatment and recovery approaches. Although recycling is a commonly adopted method, pyrolysis and gasification are potential options. Pyrolysis is regarded as a promising route towards a circular and sustainable economy that deals not only with EOLTs but also with different plastic wastes [32]. EOLTs typically contain about 90% organic matter and possess a high heating value (HHV) ranging from 29 to 39 MJ/kg. The pyrolysis process is a thermochemical depolymerisation process that has attracted significant interest due to the unique properties of the products it generates. Therefore, the recovery of energy and valuable by-products from EOLTs through pyrolysis is desirable and promising [33,34,35].

The pyrolysis process involves heating EOLTs in an inert atmosphere at temperatures typically ranging from 400 to 600 °C. This process breaks down and separates the organic components of the tyres. The products of pyrolysis, often referred to as tyre-derived products (TDPs), include scrap steel (about 15 wt% of the output, extracted during pretreatment), char (30–40%), oil (40–50%), and syngas (10–20%). However, the yield of TDPs can vary depending on the operating conditions of pyrolysis [36,37,38]. The kind of reactor type, temperature, heating rate, and residence time, among other factors, influence the proportion of TDPs. Understanding the trade-offs between these factors is crucial for successfully carrying out pyrolysis.

Some of the commonly used pyrolysis processes include slow pyrolysis, fast pyrolysis, flash pyrolysis, vacuum pyrolysis, pressurised pyrolysis, microwave pyrolysis, plasma pyrolysis, hydrogenation pyrolysis, co-pyrolysis, and catalytic pyrolysis [39]. The mechanisms that enhance the extent of production of pyrolysis products differ significantly among these technologies. For instance, slow pyrolysis operates at lower temperatures and longer residence times, making it more suitable for producing char. On the other hand, fast pyrolysis involves rapid heating to moderate temperatures, optimising oil yield. In contrast, flash pyrolysis employs extremely rapid heating to high temperatures, resulting primarily in gases. These varying conditions lead to different yields of char, oil, and gas.

Each pyrolysis technology improves the products through distinct mechanisms. For example, vacuum pyrolysis develops negative pressure, rapidly removing volatiles from solid wastes and reducing their residence time in the pyrolysis reactor. Conversely, pressurised pyrolysis inhibits the diffusion of volatiles, prolonging their residence time and increasing the probability of secondary cracking reactions. In microwave pyrolysis, the material absorbs microwave energy and converts it to heat energy, leading to the pyrolysis of waste tyres. Plasma pyrolysis utilises an electric current that passes through the gas to generate a continuous arc that promotes the secondary cracking of volatiles. Hydro-pyrolysis helps regulate pyrolysis volatiles to form more saturated compounds. Co-pyrolysis can take advantage of two materials with different compositions, which complement each other and exhibit a synergistic effect, thus improving the quality of the pyrolysis products. In catalytic pyrolysis, catalysts help lower the activation energy of the pyrolysis process, enabling rapid conversion of waste tyres [7,40].

Pyrolysis reactors play a crucial role in breaking down raw material under controlled conditions involving high temperatures, fluctuating pressures, and an absence of air with inert or fluidised gases. Different reactors are used to attain the necessary parameters needed to maximise the production of desired outputs, such as oil, char, or gas. Pyrolysis reactors can be classified based on the movement of the feedstock inside them. There are stationary feedstock reactors, where the feedstock does not move, and movable feedstock reactors, where the feedstock is set in motion using additional forces. Pneumatic forces are often used to transport the feedstock in different reactors, including fluidised bed reactors, spouted bed reactors, circulating bed reactors, and vortex reactors. Additionally, the feedstock can be circulated within the pyrolyser by mechanical means such as augers, stirrers, ablative mechanisms, screws, centrifuges, and rotary motors.

Rotary kilns are cylindrical vessels that rotate to facilitate even heating. Fixed-bed reactors consist of a stationary bed where the feed is placed. Fluidised bed reactors enhance mixing and heat transfer by fluidising feed particles. Screw reactors transport the feed through a heated chamber using a screw conveyor. Therefore, selecting the appropriate pyrolysis type, reactor type, and control of operational parameters such as temperature, heating rate, and residence time are essential to maximise the product yields [41,42].

Some of the benefits of pyrolysis of EOLTs include

1. A 90% reduction in human toxicity potential (HTP) and ozone layer depletion potential (ODP);
2. An 84% reduction in abiotic depletion potential (ADP) of fossil fuels and minerals;
3. A reduction of 2.5 kg of CO_2_ emissions per kg of virgin carbon black produced [5].

### Challenges and Considerations in Scaling up EOLT Pyrolysis

Although pyrolysis is increasingly recognised as a viable solution for managing EOLTs compared to traditional methods like landfilling and incineration, its widespread large-scale deployment remains limited. Several pilot and industrial demonstration plants already operate; however, broader commercial adoption is still in the conceptual development phase due to economic, regulatory, and technical challenges. To scale up pyrolysis facilities effectively, the following key issues must be addressed:

- Standardisation of pretreatment procedures: Establishing a standard pretreatment procedure for EOLTs is essential to achieve the optimal feedstock size for the pyrolysis reactor and ensure consistency in the output quality.
- Upgrading TDPs: Developing processes to upgrade the TDPs, especially oil and rCB, to establish stable and profitable markets.
- Supply chain coordination: Ensuring a steady supply of tyres is complicated due to the need to coordinate collection from multiple locations and manage the associated transportation costs.

In addition, several important factors that must be considered to ensure the safe operation of large-scale pyrolysis plants are

- Obtaining necessary work approvals and environmental permissions;
- Creating detailed commissioning and operating guidelines;
- Providing adequate training for staff members;
- Implementing robust engineering designs to address hazards, such as conducting HAZOP (Hazard and Operability) studies [30,38].

## 4. Pyrolysis of Tyre-Derived Products

Pyrolysis has become a prominent recycling method, which meets the three principles of solid waste treatment: reduction, resource recovery, and fewer pollutants. Plus, it produces four valuable products, steel, oil, char, and gas, often referred to as tyre-derived products (TDPs) [7].

### 4.1. Scrap Steel or Steel Wire

Steel wire can be part of TDPs at up to 15 to 20 wt%. It can be recovered from EOLTs either before or after the pyrolysis process, depending on the configuration of the recycling plant. The quantity of steel wire recovered varies based on the type of tyre, with OTR tyres containing a higher steel content. Once the steel is reclaimed, it undergoes further processing to produce commercial-grade billets. A significant portion of recycled steel is used to make new steel, contributing to the expansion of the global scrap steel market as industries strive to reduce their carbon footprints [43,44].

### 4.2. Syngas or Tyre-Derived Pyrolysis Gas (TPG)

TPG constitutes approximately 10–20% of the product yield on a steel-free basis and 8–17% when steel is included. The primary components of TPG are hydrogen (H_2_), methane (CH_4_), and alkanes and alkenes (C_2_ to C_4_). It also contains minor amounts of hydrogen sulphide (H_2_S), carbon monoxide (CO), and carbon dioxide (CO_2_). Due to its high calorific value, TPG is used for heating reactors. Additionally, it can be utilised in gas engines to produce electricity or be supplied to boilers.

### 4.3. Oil or Tyre-Derived Pyrolysis Oil (TPO)

TPO (or Tyre Pyrolysis Oil) is a thick, dark brown or black, viscous liquid. It is the primary product of EOLTs’ pyrolysis process, constituting approximately 40–50% on a steel-free basis and 35–45% with steel. TPO contains hydrocarbons, aromatic compounds, and organic compounds. Due to its high density and viscosity, as well as its oxygen, sulphur, and nitrogen content, TPO cannot be used directly as diesel. Refining processes such as distillation, hydrotreating, and desulphurisation are required to upgrade TPO to meet the environmental standards [45]. Market options for TPO include its use as a fuel to generate electricity for large marine engines, as a substitute for bunker oil, and as a feedstock for BTEX (Benzene, Toluene, Ethylbenzene, and Xylene) chemicals [46]. An emerging application of TPO involves the production of sustainable carbon black (sCB), a topic that will be explored further in the following section.

### 4.4. Char or Tyre-Derived Pyrolysis Char (TPC)

Also known as tyre pyrolysis carbon, TPC is obtained from the pyrolysis of shredded steel-free tyres, yielding 30–40% of products. When whole tyres with steel are processed, the yield ranges from 25 to 35% of products. TPC is a carbon-rich solid residue [38,47]. It is free of wire and fabric and is called recovered carbon black or rCB. More than 80% of rCB is carbon black. It also contains inorganic compounds such as ash, silica, zinc, and sulphur [48]. Due to high ash content and the presence of impurities compared to commercial carbon black (CB), the applications of rCB are limited compared to the other TDPs. Some of the applications of rCB include its use as a solid fuel, adsorbent (activated carbon), reinforcing agent for tyre rubber, materials for batteries and capacitors, printing ink, and as a filler in asphalt modification [7,27,49].

**Figure 4:**
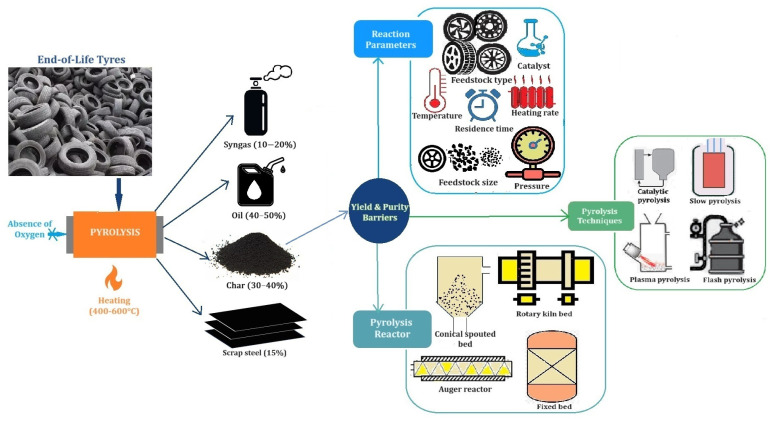
End-of-life tyres pyrolysis: factors affecting yield and purity of rCB (refined char). Note: The reported yields of pyrolysis products vary widely depending on tyre composition and operating conditions. The ranges cited (e.g., 15% steel, 40–50% oil, 30–40% char, and 20% gas) originate from different studies and are not intended to yield a sum of 100%. Variations arise because steel is often reported separately from the organic fractions. Also, different experimental conditions lead to different product distributions. The influence of pyrolysis parameters on rCB yield and purity is, approximately, as follows: temperature (30–40%), heating rate and residence time (10–20%), catalysts (5–10%), and feedstock size and system pressure (each less than 10%). These weighted estimates are based on various experimental and techno-economic studies and may vary with reactor type and feedstock characteristics. Further standardised research is required to refine these values.

For over two decades, extensive research has been conducted on EOLT pyrolysis, and many authors have reviewed this information regarding various pyrolysis reactors, technology, products, their properties, and applications. Additionally, the effects of operating parameters like particle size (whole, shredded, granules, crumbed tyres, or belts), tyre type (passenger, truck, OTR, conveyor belt, etc.), catalyst, temperature, heating rate, pressure, and residence time were investigated [7,39,40,50]. Based on these reviews, the parameters that influence the yield and quality of rCB can be summarised as shown in Figure 4.

These findings highlight the complex decision-making in selecting the appropriate reactor design, pyrolysis technique, and process variables. Also, the choices depend on the desired outcomes’ specific quantity and quality requirements. Moreover, each option presents unique advantages and limitations, emphasising the challenges of optimising the pyrolysis process.

In addition to considerations of overall yield and product distribution, it is also important to account for how individual reactor design configurations and operating conditions affect the microstructure and surface properties of rCB. For example, in fluidised bed reactors, the fluidisation velocity can have a profound impact on surface area and pore development of rCB, particularly in the activation portion of the process. Min & Harris noted that increasing the fluidisation velocity (up to 4× the minimum fluidisation velocity) resulted in increases in BET surface area and total pore volume to 1011 m^2^/g and 1.56 cm^3^/g, respectively [51]. Jin et al. studied the fluidisation number and its effect on SBET (specific surface area), noting that its value decreased with increasing velocity when the temperature was 550 °C, but increased when the temperature was 650 °C. The implication is that there is an interaction between thermal and hydrodynamic conditions that impact agglomeration, devolatilisation, and subsequent particle development [52]. Furthermore, fixed-bed reactors usually have longer residence times and poorer heat transfer, which can promote secondary reactions that may increase ash content and alter surface chemistry [7].

Rotary kilns, characterised by tumbling actions and heterogeneous thermal zones, will tend to yield rCB with a bulkier particle size distribution (PSD) and less uniform morphology [53]. The temperature, residence time and reactor mixing affect the bulk properties of the rCB, such as ash content, PSD, and surface functionalities; however, systematic studies on the influence of fluidisation velocity of tyre pyrolysis on microstructural features are lacking. Thus, the literature gives some confidence but also demonstrates the need for more reactor-focused studies if rCB quality is to be optimised.

## 5. New Classification of Carbon Black

Commercial carbon black (CB) or virgin carbon black (vCB) is one of fifty essential industrial chemicals produced globally, with an annual yield of 8.2 billion kilograms (or 8.2 million metric tonnes). It comprises over 95% carbon and is primarily produced using furnace black and thermal black processes. However, the production of vCB is resource-intensive, requiring 1.5 kg of fossil fuels and significant amounts of water for every kilogram manufactured. Furthermore, it emits 1.5 to 2.5 kg of CO_2_ for each kg produced [54,55,56].

In contrast, recovered carbon black (rCB), which is derived from end-of-life tyres (EOLTs), offers a more sustainable alternative. The production of rCB emits five times less CO_2_ than vCB production. Specifically, while vCB produces approximately 10 kg of CO_2_ for every kilogram manufactured, rCB generates only about 2 kg, resulting in an 80% reduction in carbon footprint. It is also 15–30% less expensive, making it an attractive and cost-effective choice for industries looking to reduce carbon emissions [14]. While the commonly cited cost advantage of rCB production is based mainly on unit raw material prices, it does not fully capture the complete life-cycle costs associated with its production and use. A comprehensive life-cycle cost (LCC) assessment should include additional factors such as the collection and transportation of EOLTs, the capital investment in pyrolysis plants, operational energy and labour costs, post-treatment modifications (such as demineralisation and activation), composite processing, and the potential end-of-life management of rCB-based products.

For example, bin Samsuri reported that rCB can be up to 40–50% cheaper than certain commercial CB grades in rubber compounding, based on material price comparisons alone [57]. Similarly, the field study by Karagiannidis & Kasampalis documented EOLT collection and transport costs ranging from EUR 0.057 to over EUR 0.130 per kilogram in Greece, depending on region, logistics, and access [58]. Goksal provides a techno-economic simulation showing that the total processing cost of EOLT pyrolysis can vary from approximately EUR 0.091 to EUR 0.308 per kilogram, with net profits remaining narrow unless tipping fees or energy recovery are factored in [59].

Furthermore, rCB requires post-treatment steps (e.g., acid washing, surface functionalization) to match vCB performance in some applications, and these processes introduce additional costs. Comparative environmental life-cycle assessments, such as those by Maga et al., support the environmental benefits of pyrolysis-derived rCB, but do not evaluate composite-level economic performance [34]. Taken together, the actual economic benefit of rCB is highly dependent on factors such as plant scale, feedstock logistics, process efficiency, co-product utilisation, and downstream modification costs. Therefore, future studies should aim to establish a full LCC model that incorporates both upstream and downstream costs when comparing rCB- and vCB-based composites.

However, rCB is not a simple, direct substitute for vCB. Because of its heterogeneity, rCB differs significantly from vCB in terms of its ash content, particle size, and morphology. It also consists of a mixture of different grades of vCB. After undergoing processes such as demineralisation and surface modification, the properties of rCB can vary significantly, making it not easily comparable with vCB in certain applications. As a result, it should be considered a distinct material in its own right rather than as a one-to-one replacement for vCB [60]. A summary of several widely used modification techniques for treating tyre char (or refined rCB), aimed at reducing impurities and improving its properties, is presented in Table 1. These methods include chemical, thermal, and surface functionalisation processes, which result in significant improvements such as reductions in ash content, increases in surface area, and enhancements in mechanical performance.

Given these complexities, a new typology for carbon black is necessary. This typology should consider both the unique properties of rCB—such as its environmental advantages (lower CO_2_ emissions and cost savings) and its inherent variability—as well as its potential to support global sustainability goals. The current broad categorisation of carbon black fails to adequately address the different environmental impacts, material properties, and applications. By establishing a new typology, the material can be better aligned with the global efforts towards sustainable industrialisation and climate action.

This work developed a new typology for CB that aligns with two international agreements established by United Nations members: (1) the 2030 Agenda for Sustainable Development, which has 17 Sustainable Development Goals (SDGs), and (2) the Paris Agreement on Climate Change, signed in December 2015 [68]. The typology proposes a classification that recognises the distinct material properties of rCB and environmental aspects related to its production. It also acknowledges that rCB is not just a replacement for vCB, but rather a new material with its own challenges and opportunities. The new typology focuses on three critical SDGs:

1. Goal 9: Build resilient infrastructure, promote inclusive and sustainable industrialisation, foster innovation to deal with environmental challenges, enhance sustainability in industrial practices, and reduce waste.

By recognising rCB as a distinct material, the typology encourages industries to innovate and adopt more sustainable practices, promoting a circular economy.

2. Goal 12: Ensure sustainable consumption and production patterns.

The typology advocates for the use of recycled materials, such as rCB, reducing the demand for vCB and supporting more sustainable production and consumption practices.

3. Goal 13: Take urgent action to combat climate change and its impacts. This classification can be an extension of a future-based classification.

By identifying and categorising the reduced CO_2_ emissions from rCB production, this typology highlights opportunities for emissions reductions, directly contributing to climate change mitigation and supporting the global shift towards low-carbon industrial processes.

**Figure 5:**
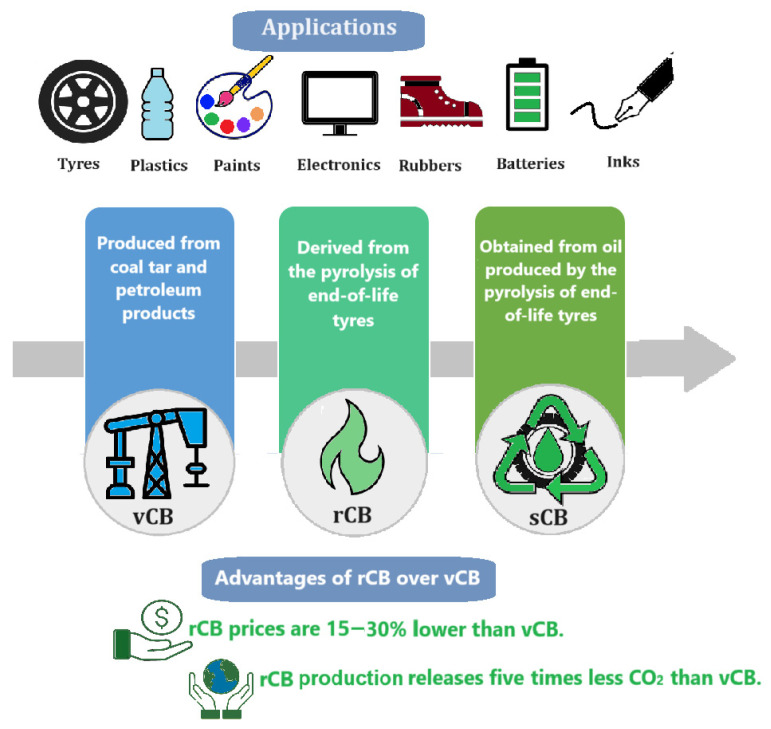
Types of carbon blacks.

This framework can serve as a foundation for future classifications of CB based on raw materials and guide the industry toward more environmentally responsible and innovative approaches to CB production. It categorises CB into three types: Figure 5 shows this future-oriented classification.

1. Virgin carbon black (vCB), also known as traditional or commercial CB.
2. Recovered carbon black (rCB).
3. Sustainable carbon black (sCB).

### 5.1. Virgin Carbon Black (vCB)

vCB, simply known as carbon black (CB), is a form of para-crystalline carbon with a high surface area-to-volume ratio. It is produced through the incomplete combustion of gaseous or liquid hydrocarbons derived from fossil fuels such as coal, petroleum, and natural gas under controlled conditions [69]. CB primarily enhances the physical and mechanical properties of rubber products, most notably in tyres. Additionally, it serves as a pigment and UV stabiliser and can function as either a conductive or insulating agent in various applications [48]. Around 70% of CB is utilised in the tyre industry, 20% in other rubber products, 9% in printing inks, coatings, and plastics, and 1% in other applications [38,56].

### 5.2. Recovered Carbon Black (rCB)

rCB is a refined form of a solid residue known as char. It is produced through the pyrolysis of EOLTs or other rubber waste. It contains approximately 80% carbon black and a mixture of inorganic compounds such as ash, silica, zinc, and sulphur [48]. It serves multiple purposes, such as a filler, pigment, and adsorbent for dyes, organic compounds, and heavy metals. In addition, rCB can be used as a supercapacitor, a conductive additive in sodium and lithium batteries, as well as a catalyst, and a precursor for nanomaterials [27].

### 5.3. Sustainable Carbon Black (sCB)

sCB is the CB produced from oil derived through the EOLT pyrolysis process using a furnace reactor. The BlackCycle consortium has announced the world’s first production of sCB, which possesses the same properties as conventional CBs for tyre applications. Orion Engineered Carbons, a consortium partner, has successfully produced two types of sCBs. These carbon blacks, named sN234 and sN347, are direct substitutes for the conventional CB types N234 and N347 [38,70].

## 6. Composite Materials

Materials serve as the backbone of the manufacturing sector, offering a wide range of options from elemental metals to composite structures. Composite materials have been widely used in modern manufacturing, significantly replacing traditional materials due to their exceptional properties, like high specific strength, robust damping capabilities, and an increased specific modulus [71].

A composite material consists of two phases: the continuous phase, usually known as the matrix or base phase, and the discontinuous phase, the reinforcing phase, or filler materials. These phases work at a macroscopic level to enhance the overall properties of the composite material. Composite materials are characterised by the heterogeneous amalgamation of two or more materials, each with distinct morphologies, compositions, and properties. The final composite material typically exhibits different and, often, superior properties compared to its individual constituents [72].

Reinforcement in composite materials plays a crucial role in resisting applied mechanical loads. To effectively resist these loads, reinforcement materials are usually chosen for their hardness, brittleness, and tensile strength. Depending on the shape and size of the reinforcement, it can be classified into two main types: particulate reinforcement and fibre reinforcement [73]. The matrix surrounds and binds the reinforcement, providing protection and reinforcement.

Composite materials can be further classified based on the type of matrix used, which includes metal matrix composites, ceramic matrix composites, and polymer matrix composites. The basic properties of these groups are high strength and rigidity, especially in ceramic and metal composites, while polymer composites tend to have lower density but increased strain capacity [71]. The polymer matrix can be thermoplastic, thermosetting, or elastomer-type.

### 6.1. Evolution of Reinforcements in Polymer Composites

Polymers have gained popularity because of their low density, making them suitable for various applications, such as automotive, construction, aerospace, and domestic appliances. However, polymers often need to be augmented to meet operational requirements, especially in severe environments such as high mechanical loads and intense friction and wear.

Experimental studies have confirmed the fact that adding reinforcements or filler materials can significantly improve the thermomechanical, dielectric, and other essential properties of polymer composites [73]. Comprehensive studies have been conducted to analyse the application of several micro-fillers, such as glass and aramid (commercially known as Kevlar), and nano-fillers, such as carbon nanotubes and graphene. These studies have also included a variety of fibres, including natural and synthetic ones, as fillers. The natural fibres explored include (1) plant-based fibres such as bamboo, banana, palm, coir, sisal, agave, screw pine, cotton, ramie, hemp, jute, munj, sikki, benakati, and madhurkati, (2) animal-based fibres such as wool, feathers, hairs, and silk, and (3) mineral-based fibres like asbestos, sepiolite, basalt, and mineral wool. Synthetic fibres made of carbon, glass, graphite, aramid, silicon carbide, and boron have also been used [74].

Based on the studies reviewed, the most frequently utilised synthetic fibre composite exhibits excellent mechanical properties and cost-effectiveness. However, there is a growing trend towards natural fibres due to economic production, environmental awareness, and sustainability. Natural fibres are lightweight, cost-effective, and environmentally friendly, with high impact resistance and flexibility, making them particularly suitable for the automotive and electronics industries to reduce fuel consumption and lower emissions. Despite problems such as moisture resistance, aggregation during production, and specific thermal limits, research has continued to optimise the performance of these materials. Efforts have also been made to explore hybrid and carbon-based polymer composites, with particular focus on those produced from the combination of natural–synthetic and natural–natural fibres, studying predominantly their tensile and flexural properties as well as their chemical resistance [71,75].

There has been a growing interest in the study of polymer composites in recent years. One of the exciting research areas involves conductive polymer composites, which include particular types of polymers known for their enhanced electrical conductivity. These include polyaniline, polythiophene, polyacetylene, polypyrrole, poly (phenyl vinylene), poly(p-phenylene), and PEDOT or poly(3,4-ethylenedioxythiophene). These polymers are widely known as conjugated polymers. Another significant area of research focuses on tribological polymer composites such as polyetheretherketone (PEEK), polytetrafluoroethylene (PTFE), high-density polyethylene (HDPE), and poly(ethylenimine) (PEI). Additionally, ongoing studies are exploring polymer electrolytes, including poly(acrylonitrile) (PAN) and poly(acrylonitrile-co-butadiene) (PAB) [74].

### 6.2. Carbon-Based Polymer Composites

Carbon is widely used as a filler in polymers (thermoplastics, thermosetting polymers, and elastomers or rubbers) to improve their mechanical, thermal, and electrical properties. It is used in several forms in polymer composites, including carbon black (CB), graphite, fullerenes, nanodiamonds, carbon fibres (CFs), carbon nanofibers, carbon nanotubes (CNTs), and graphene [76,77]. The reinforcement CB provides is a function of its surface area, surface activity, and structure. Polymer chains adsorb onto the CB surface through chemical and physical interactions, restricting the movement of polymer molecules and thereby providing reinforcement. A larger surface area and increased surface activity lead to more interactions between the polymer and the filler. The surface activity of CB is a function of the type and quantity of the oxygen-containing chemical groups present on its surface, such as carboxyl, quinone, phenol, and lactone, as well as the number of graphitic crystallite ends. CB structure is understood by the size and shape of aggregates and their distribution, which further affects reinforcement. In rubber composites, the aggregate nature of reinforcing fillers primarily influences their ability to occlude rubber, effectively shielding it from deformation, whereby the occluded rubber acts as an additional filler rather than simply being part of the polymer matrix. In summary, the higher the surface area, surface activity, and structural complexity of a filler, the greater its reinforcing potential will be [78,79].

Over the past decades, different forms of carbon material have been studied as fillers in polymer composites. da Luz et al. investigated the inclusion of graphene into natural fibre polymer composites, exploring its potential and performance [80]. Mohd Nurazzi et al. studied CNT–polymer composites and their applications across various sectors, including electronics, automobiles, textiles, aerospace, sports equipment, sensors, energy storage devices, and filters [81]. Harussani et al. discussed studies on carbon-based material-reinforced composites, emphasising applications in defence and many engineering disciplines [82]. Shahamatifard et al. investigated the development and applications of carbon-based rubber nanocomposites, detailing their unique features and potential uses [83]. Geier et al. conducted a study involving advanced cutting tools and technologies for edge-trimming carbon fibre-reinforced polymer (CFRP) composites [84]. This study also highlighted the need to identify further research opportunities in the edge trimming of CFRP composites, which are increasingly in demand in the aerospace, automotive, construction, and sporting goods industries due to their lightweight and high-strength [85].

### 6.3. Scope of rCB/Polymer Composites

Carbon black (CB) is an exceptional filler for polymer composites due to its distinctive properties. However, its production cost is high because it is obtained from crude oil, a fossil fuel. One of the most efficient and sustainable methods to produce CB is by using waste tyres through pyrolysis. Historically, CB was produced from the pyrolysis of agricultural waste materials, such as coconut shells, oil palm shells, and bamboo stems [86]. This shift to alternative sources is driven by the imperative need for sustainable and eco-friendly practices to support the ongoing decarbonisation efforts and the goal of achieving zero carbon emissions. These efforts aim to reduce reliance on non-renewable fossil resources, minimise costs, and diminish carbon footprints [47].

Many studies have investigated the effectiveness of rCB as a filler in polymer (rubber) composites using various analytical techniques. These techniques include elemental and proximate analyses, X-ray fluorescence (XRF), X-ray diffraction (XRD), Fourier transform infrared (FTIR) spectroscopy, scanning electron microscopy (SEM), Brunauer–Emmett–Teller (BET) analysis, and particle size distribution (PSD) [87,88,89,90,91].

Studies have shown that factors such as small surface area and large primary particle sizes significantly affect conventional fillers’ reinforcing potential, such as commercial carbon black (CB). Additionally, surface activity plays a crucial role in the mechanical properties of rubber composites. This activity is associated with the distribution of highly active sites located on the surface of the filler. Consequently, several critical factors determine the surface activity of rCB, including the presence of carbonaceous residues, inorganic compounds, acidic functional groups, and the size of the primary particles, all of which influence the specific surface area (SBET). It is worth noting that the reinforcement properties of the rCB are lower than those of commercial CB. However, this does not imply that rCB is unsuitable for rubber compounding; it simply indicates that it cannot match the behaviour of commercial CB when comparing the properties of the resulting composites using the same loading levels. Therefore, one of the main challenges associated with incorporating rCB into rubber formulations is finding a balance between loading and the final properties of the composite [79].

Research has shown that untreated tyre char (rCB) can potentially replace commercial CB in rubber additives, offering significant recycling potential. Table 2 shows the improved characteristics of various polymer composites that incorporate rCB as a filler. Some studies have also compared the rCB/polymer composite properties with commercial CB grades. As per the American Society for Testing and Materials (ASTM D1765), the letter “N” denotes a normal curing rate, the first digit designates the average surface area of the carbon black as measured by nitrogen surface area, and the third and fourth characters in this system are arbitrarily assigned digits [92]. For instance, Ismawi et al. examined mechanical properties with N774 and N660; Norris et al. analysed performance properties with N772, N550, N330, N234, and N115; Berki et al. focused on mechanical properties with N330; Karabörk and Tıpırdamaz assessed curing properties, tensile strength, elongation at break, modulus, tear strength, hardness, and dynamic mechanical properties with N550; Sagar et al. explored processing and mechanical properties with N550; Dwivedi et al. characterised rCB as a substitute for N330; and Urrego-Yepes et al. observed rheological, thermal, structural, and mechanical properties with N550. These studies [78,79,87,88,89,93,94] collectively demonstrate that rCB can potentially replace CB if the pyrolysis process is optimised to enhance rCB quality to be comparable to CB. Additionally, incorporating CB along with rCB in rubber composites can achieve the desired properties for industrial use. However, a major drawback of rCB is its high residual ash and sulphur content, which necessitates its modification and purification. The main problem with the high ash content of tyre char stems from the addition of minerals during manufacturing, which limits the possible end uses of the material. In addition, the presence of ash and volatile oily matter can obstruct pores during pyrolysis, further compromising the quality.

The above discussion demonstrates that the advantages, such as sustainability, cost, and recycling potential, and disadvantages, such as lower reinforcement, ash and sulphur content, and variability of rCB composites relative to conventional CB, have already been critically assessed in this section. RCB is generally suitable for many applications, but unacceptable shortcomings occur when it is used as a direct one-to-one replacement for high-performance CB grades without optimisation. Due to differences in pyrolysis conditions, polymer matrices, loading levels, and benchmarking standards across the studies discussed, direct comparison of rCB performance with that of traditional materials is challenging. Future work should establish standardised testing conditions or adopt normalisation protocols to facilitate more rigorous cross-study comparisons. These trade-offs are further contextualised in the following SWOT analysis and within the proposed vCB–rCB–sCB classification, representing this review’s key innovation points.

## 7. SWOT Analysis

The advantages and disadvantages of using rCB as reinforcement in polymers are outlined in a SWOT analysis, as presented in Table 3 [14]. This analysis identifies the strengths and opportunities of rCB reinforcement in polymers, drawing on the relevant literature. Additionally, it examines the weaknesses and threats to highlight the challenges and limitations that may arise. This comprehensive analysis will aid in making informed decisions regarding the technological adoption and optimisation of rCB polymer reinforcement applications, considering various economic and environmental factors.

This SWOT analysis provides a valuable framework for organisations planning to establish pyrolysis plants or those that already operate existing facilities. It helps identify strengths, weaknesses, opportunities, and threats to develop a path for comprehensive analysis. As a result, economic outcomes can be improved without compromising environmental sustainability. This approach promotes sustainability and aligns with the principles of the circular economy. Therefore, it ensures that the integration of rCB into polymer reinforcement can be performed efficiently and responsibly.

## 8. Conclusions and Future Directions

Among all waste management practices, pyrolysis has proven to be a sustainable solution for managing end-of-life tyres (EOLTs). It addresses solid tyre waste utilisation and produces four valuable secondary products with minimal environmental impact. This review focuses on recovered carbon black (rCB) obtained from pyrolysis of EOLTs, demonstrating properties comparable to commercial carbon black (CB) and potential as a polymer reinforcement.

The review summarises previous research on the reinforcing capacity of rCB in various polymers and compares it with those of commercial CB (N330 and N550). Building on these studies, the current work proposes a new classification system for carbon blacks and includes a SWOT analysis of using rCB as a reinforcement in polymer composites. The SWOT analysis is intended to guide organisations to understand the benefits and challenges that must be addressed before adopting this material in their applications.

In conclusion, rCB has shown the potential to replace some commercial grades of CBs partially. A greater emphasis on this area may position rCB as a viable substitute for CB in polymers. Therefore, future research should focus on establishing standard procedures to measure the quality of rCB and to optimise pyrolysis conditions. This optimisation would enhance its purity by reducing ash content and impurities, which currently limit the rCB market for commercial applications.

Furthermore, it is evident from many studies that many factors influence the pyrolysis of EOLTs, including technology, equipment, operating conditions, and the composition of the tyre feedstock. Pressurised pyrolysis shows promise for producing high-quality oil and char product yields while emitting fewer pollutants. However, this method is still mainly in the research phase and needs further investigation. Furthermore, most current research on tyre char is in its early stages and often needs more detailed technical and economic analysis of its commercial viability. Addressing these research gaps will help the development of fossil fuel-free CB, which can be used as a reinforcement in developing polymer composites, leading to greener and more sustainable materials.

In addition to shortened technical optimisation, interdisciplinary innovations and areas of emerging application are expected to improve the utility of rCB further. Advanced tools such as artificial intelligence (AI) and machine learning (ML) can support real-time control of pyrolysis parameters, like reactor temperature and residence time, to improve yield and quality consistency. ML models also offer the potential to predict the mechanical and thermal behaviour of rCB/polymer composites, accelerating material design and reducing experimental effort.

On the application side, rCB is increasingly used as a sustainable filler in rubber and thermoplastic composites. With appropriate surface modification and blending strategies, its performance can approach that of vCB. These interdisciplinary advances contribute to more tailored, high-performance composites and strengthen the case for rCB in industrial polymer applications.

## Figures and Tables

**Table 1:** Literature-based summary of rCB/tyre–char modification methods.

Treatment Method	Key Effects	Quantitative Outcomes	Source
Molten salt thermal treatment (Na_2_CO_3_–NaOH)	Removes inorganic ash and sulphur; breaks agglomerates; improves particle uniformity	>80% ash removal and >70% S removal at ≈400 °C; 71–83% char recovery	[61]
Acid washing—HNO_3_ (5 M)	Demineralisation; increases surface acidity (–COOH); preserves pore structure	Increased carboxyl (–COOH) groups on the char’s surface enhanced its acidity by 57.8% and reduced the sulphur content	[37]
Acid washing—HCl (rinsing)	Demineralisation; lowers ash; improves mechanical properties in rubber compounds	Ash content reduced from 22.5% to 8.4% and tensile stress increased by ≈2.2 MPa at 300% strain.	[62]
Acid/alkali sequential demineralisation (HNO_3_ + NaOH, etc.)	Strong ash reduction; improves BET and particle size after milling	Ash content reduced from 16.24% to 1.95%; BET surface area increased from 58.9 to 80.22 m^2^/g; particle size decreased from 153.2 to 38.5 µm	[63]
Thermal activation (CO_2_ and steam at high temperature 900 °C)	Develops porosity and raises the BET strongly	Steam activation improves carbon conversion by 30–70% over CO_2_; surface area: steam 666.6 m^2^/g; CO_2_ 434.5 m^2^/g	[64]
Chemical activation (KOH, CO_2_) and Acid treatments (HNO_3_/H_2_SO_4_ & HNO_3_/H_2_O	Creates microporosity; achieves very high BET surface area	Acid treatments removed ~70% of impurities and increased the BET surface area to 38–57 m^2^/g; the BET surface area is 720 m^2^/g after CO_2_ activation and 242 m^2^/g after KOH activation.	[65]
Plasma treatment (O_2_, Ar glow discharge)	Adds oxygen-containing groups; improves dispersion and mechanical properties	O/C ratio and XPS O-peak intensities increased 10–30%; fatigue/wear resistance improved by 15–25%	[66]
Silane grafting/coupling agents (e.g., 3-aminopropyltriethoxysilane)	Improves bonding with polar polymers (PP, silica/CB blends); restores mechanical properties	Up to 300% increase in tensile strength and modulus compared to untreated char	[67]
Graft polymerization/covalent functionalisation (–COOH, –NH_2_ grafts)	Adds functional groups; improves compatibility; reduces hysteresis in elastomers	Mechanical recovery and dispersion improved by approximately 10–40%	[27]

**Table 2:** Analysis of the impact of rCB reinforcement on polymer composite performance.

rCB Name in Paper	Polymer Matrix	Properties Analysed	Compared Commercial CB Grades	Reference
Recovered carbon black (rCB)	Nitrile butadiene rubber (NBR)	Tensile properties and curing characteristics	N774 and N660	[93]
Pyrolytic carbon black (PCB)	Vulcanised rubber	Extend stress, tensile strength at break, and elongation ratio	-	[95]
Pyrolysis carbon black (pCB)	Styrene butadiene rubber (SBR)	Elastic modulus and filler dispersion	N772, N550, N330, N234, and N115	[78]
Waste tyre-derived carbon	Polyaniline (PANI)	Pseudocapacitive electrode properties like capacitance and cycle life	-	[8]
Pyrolytic carbon black (CBp)	Natural rubber (NR)/styrene–butadiene rubber (SBR) blends	Curing properties, tensile strength, elongation at break, modulus, tear strength, hardness, and dynamic mechanical properties	N550	[88]
Pyrolytic carbon black (pCB)	Styrene–butadiene rubber (SBR)	Dispersion, cure behaviour, dynamic mechanical, thermal behaviour, and tensile mechanical and fracture mechanical properties	N330	[94]
Recycled carbon black (rCB)	Ethylene–propylene–diene rubber (EPDM)	Hardness, tensile strength, elongation at break, and tear strength	N550	[89]
Pyrolytic tyre char	Polypropylene	Tensile properties, impact strength, and degradation stability	-	[90]
Recovered carbon black (rCB)	Natural rubber (NR)	Tensile strength and elongation at break	N330	[87]
Waste carbon tyres (WCTs)	Castor-based polyurethane (CPUC)	Physical, rheological, mechanical, morphological and thermal properties	-	[96]
Recovered carbon black (rCB)	Natural rubber (NR)	Rheological, thermal, structural, and mechanical properties	N550	[79]
Tyre-recovered carbon black (Tyre rCB)	Styrene–butadiene rubber	Tear strength, tensile strength, hardness, and elasticity	-	[97]
Pyrolytic carbon black (PCB)	Natural rubber	Thermal conductivity, vibration damping, and sound transmission loss	-	[91]

**Table 3:** SWOT analysis: rCB as a sustainable reinforcement in polymers.

Strengths	Weaknesses
Perception of end-of-life tyres (EOLTs) as valuable resources rather than waste. Sustainable alternative to virgin carbon black (vCB), reducing fossil resource depletion. Cost-effective and eco-friendly material derived from pyrolysis of EOLTs. Supports a circular economy and corporate sustainability goals. Versatile applications in polymers such as NBR, SBR, NR, polypropylene, etc.	Quality inconsistency due to feedstock variability and impurities (e.g., ash and silica). Lack of standardised testing and classification systems for rCB. Technological immaturity of pyrolysis processes, primarily focused on oil recovery. Challenges in the collection, transportation, and refining of EOLTs to meet demand. Lower reinforcing performance compared to commercial CB grades.
**Opportunities**	**Threats**
Increasing global focus on EOLT management and regulations to reduce stockpiles and illegal dumping. Volatility in oil prices makes rCB a competitive alternative to vCB. Development of new markets in construction, automotive, and consumer goods. Advance pyrolysis technology and standardisation efforts.	Slow industry adoption due to long qualification timelines. Competition with vCB and other sustainable fillers. Concerns about cost competitiveness * if rCB quality is not consistently improved. Regulatory hurdles and certification requirements for rCB products.

* The cost competitiveness of rCB compared to vCB depends on factors such as crude oil prices, where rCB becomes more viable when crude oil prices exceed approximately EUR 0.11 to EUR 0.13 per litre of oil, pyrolysis plant scale, with plants processing 30,000 kg/day or more reducing unit costs, and regional tipping fees for EOLTs, typically ranging from EUR 0.05 to EUR 0.10 per kg in Europe. Efficient co-product recovery also influences profitability. These factors collectively contribute to the narrow economic margins of rCB production, as reported by Goksal, Karagiannidis, & Kasampalis and Maga et al. [34,58,59].

## Data Availability

No new data were created or analysed in this study. Data sharing is not applicable to this article.

## Abbreviations

The following abbreviations are used in this manuscript:

EOLTs	End-of-life tyres
TDPs	Tyre-derived products
rCB	Recovered carbon black
CB	Commercial carbon black
vCB	Virgin carbon black
sCB	Sustainable carbon black
CAGR	Compound annual growth rate
OTR	Off-the-road
EPR	Extended Producer Responsibility
TDA	Tyre Derived Aggregate
TDF	Tyre-derived fuel
HHV	High heating value
HTP	Human toxicity potential
ADP	Abiotic depletion potential
TPO	Tyre Pyrolysis Oil
TPC	Tyre-derived pyrolysis char
SDGs	Sustainable Development Goals
PEDOT	poly(3,4-ethylenedioxythiophene)
PEEK	Polyetheretherketone
PTFE	Polytetrafluoroethylene
HDPE	High-Density Polyethylene
PEI	Poly(ethylenimine)
PAN	Poly(acrylonitrile)
PAB	Poly(acrylonitrile-co-butadiene)
CF	Carbon fibres
CNTs	Carbon nanotubes
XRF	X-ray fluorescence
XRD	X-ray diffraction
FTIR	Fourier transform infrared spectroscopy
SEM	Scanning electron microscopy
BET	Brunauer–Emmett–Teller
PSD	Particle size distribution
SBET	Specific surface area
ASTM	American Society for Testing and Materials
LCC	Life-cycle cost

## References

[1] Antoniou N., Stavropoulos G., Zabaniotou A. (2014). Activation of End of Life Tyres Pyrolytic Char for Enhancing Viability of Pyrolysis—Critical Review, Analysis and Recommendations for a Hybrid Dual System. Renew. Sustain. Energy Rev..

[2] IMARC Tire Market Report by Design, End-Use, Vehicle Type, Distribution Channel, Season, and Region 2024–2032. https://www.researchandmarkets.com/reports/5936421/tire-market-report-design-end-use-vehicle#product--toc.

[3] TSA Australian Tyre Consumption and Recovery—2022–2023. https://www.tyrestewardship.org.au/tools-and-resources/tyre-consumption-recovery.

[4] Genever M., O’Farrell K., Randell P., Rebbechi O. (2017). National Market Development Strategy for Used Tyres.

[5] Contec Australia Tire Waste Statistics in 2024. https://contec.tech/tire-waste-statistics-in-2024/.

[6] Kibria M.A., Thomas B.S., Bhattacharya M., Bhattacharya S. (2024). Processing of Metal-Free End-of-Life Tyres (Eolts) to Fuels and Products: An Experimental Study with Process Simulation and Economic Analysis from an Australian Perspective. Clean Technol. Environ. Policy.

[7] Gao N., Wang F., Quan C., Santamaria L., Lopez G., Williams P.T. (2022). Tire Pyrolysis Char: Processes, Properties, Upgrading and Applications. Prog. Energy Combust. Sci..

[8] Boota M., Paranthaman M.P., Naskar A.K., Li Y., Akato K., Gogotsi Y. (2015). Waste Tire Derived Carbon-Polymer Composite Paper as Pseudocapacitive Electrode with Long Cycle Life. ChemSusChem.

[9] (2025). Standard Terminology Relating to Recovered Carbon Black (rCB).

[10] Campuzano F., Martínez J.D., Agudelo Santamaría A.F., Sarathy S.M., Roberts W.L. (2023). Pursuing the End-of-Life Tire Circularity: An Outlook Toward the Production of Secondary Raw Materials from Tire Pyrolysis Oil. Energy Fuels.

[11] Afash H., Ozarisoy B., Altan H., Budayan C. (2023). Recycling of Tire Waste Using Pyrolysis: An Environmental Perspective. Sustainability.

[12] Han W., Han D., Chen H. (2023). Pyrolysis of Waste Tires: A Review. Polymers.

[13] WBCSD Managing End-of-Life Tires. https://docs.wbcsd.org/2018/02/TIP/End_of_Life_Tires-Full-Report.pdf.

[14] Pascal S., Plessis S. Evaluating the Path to a Sustainable Tire Industry: Unlocking the Potential of Recovered Carbon Black. https://www.emerton.co/news/evaluating-the-path-to-a-sustainable-tire-industry-unlocking-the-potential-of-recovered-carbon-black#.

[15] Sienkiewicz M., Kucinska-Lipka J., Janik H., Balas A. (2012). Progress in Used Tyres Management in the European Union: A Review. Waste Manag..

[16] Bansal P.N., Kapgate D.B. (2023). Tyre Waste Management: Where We Are & Where We Are Headed. Int. J. Res. Appl. Sci. Eng. Technol..

[17] Fletcher R., Wilson H. (1981). The Role of Pyrolysis in the Disposal of Waste Tyres. Resour. Conserv. Recycl..

[18] Araki T., Niikawa K., Hosoda H., Nishizaki H., Mitsui S., Endoh K., Yoshida K. (1979). Development of Fluidized-Bed Pyrolysis of Waste Tires. Resour. Conserv. Recycl..

[19] Kaksonen A.H., Gazeau B., Caceres Ruiz A.M., Cheng K.Y., Minunno R., Zaman A., Boxall N. (2024). Best Practice Case Studies for Increasing Value Recovery from End-of-Life Tyres and Conveyor Belts.

[20] Hyder V. (2012). Study into Domestic and International Fate of End-of-Life Tyres.

[21] Nkosi N., Nhubu T., Mthombeni N.H. (2025). An Inventory Analysis of Waste Tyre Generation and Management in South Africa. Waste Manag..

[22] Kay E., Laman J. (1979). A Review of Scrap Tire Disposal Processes.

[23] Eco Green Equipment The Complete History of Tire Recycling. https://ecogreenequipment.com/the-complete-history-of-tire-recycling/.

[24] Eco Green Equipment The Future of End-of-Life Tire Recycling in 2025 and Beyond. https://ecogreenequipment.com/the-future-of-end-of-life-tire-recycling-in-2025-and-beyond/.

[25] Ławińska O., Korombel A., Zajemska M. (2022). Pyrolysis-Based Municipal Solid Waste Management in Poland—Swot Analysis. Energies.

[26] Leandri P., Rocchio P., Losa M. (2020). A Swot Analysis of Innovative High Sustainability Pavement Surfaces Containing Crumb Rubber Modifier. Road Mater. Pavement Des..

[27] Costa S.M.R., Fowler D., Carreira G.A., Portugal I., Silva C.M. (2022). Production and Upgrading of Recovered Carbon Black from the Pyrolysis of End-of-Life Tires. Materials.

[28] ETRMA European Tyre and Rubber Manufacturers’ Association. https://www.etrma.org/key-topics/circular-economy/.

[29] Randell P., Baker B., O’Farrell K. (2020). Used Tyres Supply Chain and Fate Analysis.

[30] TSA Tsa Report Demystifies Pyrolysis and Gasification of Waste Tyres. https://www.tyrestewardship.org.au/news-and-insights/tsa-report-demystifies-pyrolysis-and-gasification-of-waste-tyres.

[31] Khan W., Shyamal D.S., Kazmi A.A. (2024). Management of End-of-Life Tyres in India: Current Practices, Regulatory Framework, Challenges, and Opportunities. J. Mater. Cycles Waste Manag..

[32] Cardona-Uribe N., Betancur M., Martínez J.D. (2021). Towards the Chemical Upgrading of the Recovered Carbon Black Derived from Pyrolysis of End-of-Life Tires. Sustain. Mater. Technol..

[33] Han J., Li W., Liu D., Qin L., Chen W., Xing F. (2018). Pyrolysis Characteristic and Mechanism of Waste Tyre: A Thermogravimetry-Mass Spectrometry Analysis. J. Anal. Appl. Pyrolysis.

[34] Maga D., Aryan V., Blömer J. (2023). A Comparative Life Cycle Assessment of Tyre Recycling Using Pyrolysis Compared to Conventional End-of-Life Pathways. Resour. Conserv. Recycl..

[35] Sathiskumar C., Karthikeyan S. (2019). Recycling of Waste Tires and Its Energy Storage Application of by-Products –A Review. Sustain. Mater. Technol..

[36] de Marco Rodriguez I., Laresgoiti M.F., Cabrero M.A., Torres A., Chomón M.J., Caballero B. (2001). Pyrolysis of Scrap Tyres. Fuel Process. Technol..

[37] Seng-eiad S., Jitkarnka S. (2016). Untreated and Hno3-Treated Pyrolysis Char as Catalysts for Pyrolysis of Waste Tire: In-Depth Analysis of Tire-Derived Products and Char Characterization. J. Anal. Appl. Pyrolysis..

[38] TSA Pyrolysis of End-of-Life Tyres. https://www.tyrestewardship.org.au/tools-and-resources/pyrolysis-of-end-of-life-tyres.

[39] Hoang A.T., Nguyen T.H., Nguyen H.P. (2020). Scrap Tire Pyrolysis as a Potential Strategy for Waste Management Pathway: A Review. Energy Sources Part A Recover. Util. Environ. Eff..

[40] Lewandowski W.M., Januszewicz K., Kosakowski W. (2019). Efficiency and Proportions of Waste Tyre Pyrolysis Products Depending on the Reactor Type—A Review. J. Anal. Appl. Pyrolysis.

[41] Martínez J.D., Puy N., Murillo R., García T., Navarro M.V., Mastral A.M. (2013). Waste Tyre Pyrolysis—A Review. Renew. Sustain. Energy Rev..

[42] Zerin N.H., Rasul M.G., Jahirul M.I., Sayem A.S.M. (2023). End-of-Life Tyre Conversion to Energy: A Review on Pyrolysis and Activated Carbon Production Processes and Their Challenges. Sci. Total Environ..

[43] Contect Australia Tire Recycling Pyrolysis: Pros and Cons, Explained. https://contec.tech/tire-recycling-pyrolysis-pros-and-cons-explained/.

[44] FactMR Steel Scrap Market Outlook (2023 to 2033). https://www.factmr.com/report/steel-scrap-market.

[45] Tsipa P.C., Phiri M.M., Iwarere S.A., Mkhize N.M., Phiri M.J., Hlangothi S.P. (2023). The Effect of Pre-Pyrolysis Chemical Treatment of Waste Tyre Rubber Crumbs: Comparison between Pre-Treated and Conventional Waste Tyre-Derived Oil. J. Chem. Technol. Biotechnol..

[46] Sanchis A., Veses A., Martinez J.D., Lopez J.M., Garcia T., Murillo R. (2022). The Role of Temperature Profile During the Pyrolysis of End-of-Life-Tyres in an Industrially Relevant Conditions Auger Plant. J. Environ. Manag..

[47] Contec Australia Products. https://contec.tech/products/.

[48] Waverly Carbon Recovered Carbon Black. https://www.waverlycarbon.com/carbon-black/.

[49] Xu J., Yu J., Xu J., Sun C., He W., Huang J., Li G. (2020). High-Value Utilization of Waste Tires: A Review with Focus on Modified Carbon Black from Pyrolysis. Sci. Total Environ..

[50] Williams P.T. (2013). Pyrolysis of Waste Tyres: A Review. Waste Manag..

[51] Min A., Harris A. (2006). Influence of Carbon Dioxide Partial Pressure and Fluidization Velocity on Activated Carbons Prepared from Scrap Car Tyre in a Fluidized Bed. Chem. Eng. Sci..

[52] Jin Y., Yan J., Gu J., Cen K. (2004). Configuration of Pyrolytic Chars from Waste Tires in Fluidized Bed Reactor. Huan Jing Ke Xue Huanjing Kexue.

[53] Fang H., Hou Z., Shan L., Cai X., Xin Z. (2023). Influence of Pyrolytic Carbon Black Derived from Waste Tires at Varied Temperatures within an Industrial Continuous Rotating Moving Bed System. Polymers.

[54] CORDIS-EU Recycling Shredded Used Tyres and Rubber Waste into Personalized Recovered Carbon Black to Limit Use of Fossil Fuels and Carbon Dioxide Emission. https://cordis.europa.eu/project/id/101009283.

[55] ICBA (2016). Carbon Black User’s Guide.

[56] Recovered Carbon Black Applications. https://recovered-carbon-black.com/en/.

[57] bin Samsuri A. (2022). Evaluation of Recycled Carbon Black (R-Cb) Based on Styrene Butadiene Rubber, Natural Rubber and Nitrile Rubber Compounds. Application and Characterization of Rubber Materials.

[58] Karagiannidis A., Kasampalis T. (2010). Resource Recovery from End-of-Life Tyres in Greece: A Field Survey, State-of-Art and Trends. Waste Manag. Res..

[59] Goksal F.P. (2022). An Economic Analysis of Scrap Tire Pyrolysis, Potential and New Opportunities. Heliyon.

[60] Yu J., Xu J., Li Z., He W., Huang J., Xu J., Li G. (2019). Upgrading Pyrolytic Carbon-Blacks (Cbp) from End-of-Life Tires: Characteristics and Modification Methodologies. Front. Environ. Sci. Eng..

[61] Tang H., Hu H., Li A., Yi B., Li X., Yao D., Yao H., Yuan H. (2021). Removal of Impurities from Waste Tire Pyrolysis Char Using the Molten Salt Thermal Treatment. Fuel.

[62] Zhong R., Xu J., Hui D., Bhosale S.S., Hong R. (2020). Pyrolytic Preparation and Modification of Carbon Black Recovered from Waste Tyres. Waste Manag. Res..

[63] Martínez J.D., Cardona-Uribe N., Murillo R., García T., López J.M. (2019). Carbon Black Recovery from Waste Tire Pyrolysis by Demineralization: Production and Application in Rubber Compounding. Waste Manag..

[64] Zhang J., Jones I., Zhu M., Zhang Z., Preciado-Hernandez J., Zhang D. (2021). Pore Development During Co2 and Steam Activation of a Spent Tyre Pyrolysis Char. Waste Biomass Valorization.

[65] López F., Centeno T., Rodríguez O., Alguacil F. (2013). Preparation and Characterization of Activated Carbon from the Char Produced in the Thermolysis of Granulated Scrap Tyres. J. Air Waste Manag. Assoc..

[66] Fang Y., Dong H., Hao X., Liu Y., Tang D., Zhao H., Zhou W., Sun C., Zhang L. (2024). Enhanced Fatigue Resistance of Plasma Modified Pyrolysis Carbon Black Filled Natural Rubber Composites. Appl. Surf. Sci..

[67] TRIF C.M. (2023). Rubber Composition Containing Recycled Carbon Black for Tires. International Patent Application No..

[68] United Nations The 17 Goals. https://sdgs.un.org/goals.

[69] Weibold (2024). Weibold Academy: Astm Committee D36 Advances Standards for rCB. https://weibold.com/weibold-academy-astm-committee-d36-advances-standards-for-rcb.

[70] BlackCycle Orion Engineered Carbons Is Moving Forward on the Road to Sustainability: Creation of Sn234 Able to Be Used as a Drop-in to a Conventional N234—November 2022. https://blackcycle-project.eu/orion-engineered-carbons-is-moving-forward-on-the-road-to-sustainability-creation-of-sn234-able-to-be-used-as-a-drop-in-to-a-conventional-n234/.

[71] Kerni L., Singh S., Patnaik A., Kumar N. (2020). A Review on Natural Fiber Reinforced Composites. Mater. Today Proc..

[72] Biswal T., BadJena S.K., Pradhan D. (2020). Synthesis of Polymer Composite Materials and Their Biomedical Applications. Mater. Today Proc..

[73] Rangappa S.M., Parameswaranpillai J., Siengchin S., Kroll L. (2021). Lightweight Polymer Composite Structures: Design and Manufacturing Techniques.

[74] Patel V.K., Kant R., Chauhan P.S., Bhattacharya S. (2022). Trends in Applications of Polymers and Polymer Composites.

[75] Aliyeva N., Sas H.S., Okan B.S. (2024). Revolutionizing Transportation Composite Structures: Lightweight, Sustainable, and Multi-Scale Hybrid Design through Waste Tire-Driven Graphene, Hemp Fiber, and Bio-Based Overmoulding. J. Thermoplast. Compos. Mater..

[76] Sasikumar K., Manoj N.R., Mukundan T., Rahaman M., Khastgir D. (2019). Mechanical Properties of Carbon-Containing Polymer Composites. Carbon-Containing Polymer Composites.

[77] Yang H., Gong J., Wen X., Xue J., Chen Q., Jiang Z., Tian N., Tang T. (2015). Effect of Carbon Black on Improving Thermal Stability, Flame Retardancy and Electrical Conductivity of Polypropylene/Carbon Fiber Composites. Compos. Sci. Technol..

[78] Norris C., Hale M., Bennett M. (2014). Pyrolytic Carbon: Factors Controlling in-Rubber Performance. Plast. Rubber Compos..

[79] Urrego-Yepes W., Cardona-Uribe N., Vargas-Isaza C.A., Martínez J.D. (2021). Incorporating the Recovered Carbon Black Produced in an Industrial-Scale Waste Tire Pyrolysis Plant into a Natural Rubber Formulation. J. Environ. Manag..

[80] da Luz F., Garcia Filho F., del-Río M., Nascimento L., Pinheiro W., Monteiro S. (2020). Graphene-Incorporated Natural Fiber Polymer Composites: A First Overview. Polymers.

[81] Mohd Nurazzi N., Asyraf M.R.M., Khalina A., Abdullah N., Sabaruddin F.A., Kamarudin S.H., Ahmad S., Mahat A.M., Lee C.L., Aisyah H.A. (2021). Fabrication, Functionalization, and Application of Carbon Nanotube-Reinforced Polymer Composite: An Overview. Polymers.

[82] Harussani M.M., Sapuan S.M., Nadeem G., Rafin T., Kirubaanand W. (2022). Recent Applications of Carbon-Based Composites in Defence Industry: A Review. Def. Technol..

[83] Shahamatifard F., Rodrigue D., Mighri F. (2023). Thermal and Mechanical Properties of Carbon-Based Rubber Nanocomposites: A Review. Plast. Rubber Compos..

[84] Geier N., Xu J., Poór D.I., Dege J.H., Davim J.P. (2023). A Review on Advanced Cutting Tools and Technologies for Edge Trimming of Carbon Fibre Reinforced Polymer (Cfrp) Composites. Compos. Part B Eng..

[85] Erkmen B., Bayram G. (2021). Improvement in Mechanical, Electrical, and Shape Memory Properties of the Polystyrene-Based Carbon Fiber-Reinforced Polymer Composites Containing Carbon Nanotubes. J. Appl. Polym. Sci..

[86] Ojha S., Acharya S.K., Raghavendra G. (2015). Mechanical Properties of Natural Carbon Black Reinforced Polymer Composites. J. Appl. Polym. Sci..

[87] Dwivedi C., Manjare S., Rajan S.K. (2020). Recycling of Waste Tire by Pyrolysis to Recover Carbon Black: Alternative & Environment-Friendly Reinforcing Filler for Natural Rubber Compounds. Compos. Part B Eng..

[88] Karabörk F., Tıpırdamaz S. (2016). Influence of Pyrolytic Carbon Black and Pyrolytic Oil Made from Used Tires on the Curing and (Dynamic) Mechanical Properties of Natural Rubber (Nr)/Styrene-Butadiene Rubber (Sbr) Blends. Express Polym. Lett..

[89] Sagar M., Nibedita K., Manohar N., Kumar K.R., Suchismita S., Pradnyesh A., Reddy A.B., Sadiku E.R., Gupta U., Lachit P. (2018). A Potential Utilization of End-of-Life Tyres as Recycled Carbon Black in Epdm Rubber. Waste Manag..

[90] Saul C.L. (2019). Valorisation of Pyrolytic Tyre Char as Filler in Polypropylene.

[91] Sunali S., Mago J., Negi A., Pant K.K., Fatima S. (2024). Acoustical, Vibrational, and Thermal Investigations of Pyrolytic Carbon Black Reinforced Natural Rubber Composites. Proc. Inst. Mech. Eng. Part L J. Mater. Des. Appl..

[92] Standard Classification System for Carbon Blacks Used in Rubber Products.

[93] Ismawi D.H.A., Zaeimoedin T.Z., Saad C.S.M. Recovered Carbon Black (rCB) from Waste Tyres: Effect on Mechanical Properties of Rubber Compound. Proceedings of the Conference Malaysian Science and Technology Congress (MSTC 2010).

[94] Berki P., Göbl R., Karger-Kocsis J. (2017). Structure and Properties of Styrene-Butadiene Rubber (Sbr) with Pyrolytic and Industrial Carbon Black. Polym. Test..

[95] Jie Z., Shengji W., Tianming Y., Zhengmiao X. Modified Pyrolytic Carbon Black from Scrap Tires and Its Reinforcement Performance in Natural Rubber. Proceedings of the 2011 International Conference on Computer Distributed Control and Intelligent Environmental Monitoring.

[96] Hussain N.I.A.M., Bonnia N.N., Hirzin R.S.F.N., Ali E.S., Ratim S. (2020). Preparation of Castor-Based Polyurethane Composites Filled with Waste Carbon Tyres (Wct) as Grouting Material. J. Technol..

[97] Sharma A., Sawant R.J., Sharma A., Joshi J.B., Jain R.K., Kasilingam R. (2022). Valorisation of End-of-Life Tyres for Generating Valuable Resources under Circular Economy. Fuel.

